# A tailored, dialogue-based health communication application for patients with chronic low back pain: study protocol of a randomised controlled trial

**DOI:** 10.1186/1472-6947-13-66

**Published:** 2013-06-14

**Authors:** Jörg Dirmaier, Martin Härter, Nina Weymann

**Affiliations:** 1 Department of Medical Psychology (W 26), University Medical Center Hamburg-Eppendorf, Martinistr. 52, Hamburg 20246, Germany

**Keywords:** Chronic low back pain, Randomized controlled trial, Study protocol, Patient information, Web

## Abstract

**Background:**

Chronic low back pain is a common chronic condition whose treatment success can be improved by active involvement of patients. Patient involvement can be fostered by web-based applications combining health information with decision support or behaviour change support. These so-called Interactive Health Communication Applications (IHCAs) can reach great numbers of patients at low financial cost and provide information and support at the time, place and learning speed patients prefer. However, high attrition often seems to decrease the effects of web-based interventions. Tailoring content and tone of IHCAs to the individual patient ´s needs might improve usage and therefore effectiveness. This study aims to evaluate a tailored IHCA for people with chronic low back pain combining health information with decision support and behaviour change support.

**Methods/Design:**

The tailored IHCA will be tested regarding effectiveness and usage against a standard website with identical content in a single-blinded randomized trial with a parallel design. The IHCA contains information on chronic low back pain and its treatment options including health behaviour change recommendations. In the intervention group the content is delivered in dialogue form, tailored to relevant patient characteristics (health literacy, coping style). In the control group there is no tailoring, a standard web-page is used for presenting the content. Participants are unaware of group assignment. Eligibility criteria are age ≥ 18 years , self- reported chronic low back pain, and Internet access. To detect the expected small effect (Cohen’s d = 0.2), the sample aims to include 414 patients, with assessments at baseline, directly after the first on-page visit, and at 3-month follow-up using online self-report questionnaires. It is expected that the tailored IHCA has larger effects on knowledge and patient empowerment (primary outcomes) compared to a standard website. Secondary outcomes are website usage, preparation for decision making, and decisional conflict.

**Discussion:**

IHCAs can be a suitable way to promote knowledge about chronic low back pain and self-management competencies. Results of the study can increase the knowledge on how to develop IHCAs which are more useful and effective for people suffering from chronic low back pain.

**Trial registration:**

International Clinical Trials Registry DRKS00003322

## Background

Chronic low back pain (CLBP) is a very prevalent and disabling public health problem with a significant burden on individuals, and negative social and economic effects [1]. The prevalence of CLBP is rising, the percentage of people living with CLBP more than doubled between 1992 and 2006 [2], estimates suggest that up to 12% of people experience CLBP [3] in the past 12 months. CLBP is one of the most common causes for disability [4] and results in high total expenses for the health care system [5].

Active patient involvement is a key component of effective treatments of CLBP and is equally demanded by patients, practitioners, scientists and politicians. There is a general call for more involvement in the making of medical decisions in recent clinical practice guidelines as well as for active involvement in the management of CLBP, due to important trade-offs between potential benefits, harms, costs and burdens of alternative therapies [6]. Two main aspects of patient involvement are self-management and involvement in medical decisions. Patients have to make decision regarding life style changes, medications, and use of different health services or medical interventions. Both for the involvement in medical decisions and for self-management patients need to be informed about their disease, its course, and the treatment options at hand, including their advantages and disadvantages. Providing disease-specific information to patients with CLBP e.g. can lead to better adherence to different kinds of self-management strategies [7,8]. However, due to limited resources in health care, large numbers of patients still do not have access to feasible information on CLBP.

Because of emerging Internet penetration, the ehealth tools are one current option to spread health information to a great quantity of patients with low financial burden, taking into account individual preferences with regard to time, place and previous levels of knowledge. Small but consistent effects on clinical outcomes [7-9] could be shown in trials of soundly developed online health interventions even in older populations who are expected to use the web less often [10]. Similar effects of ehealth interventions can also be shown for patients with CLBP [9-12]. There is a broad range of interactive technologies which can be used to deliver health information. Murray et al. [11] found that “Interactive Health Communication Applications” (IHCAs), a format that combines health information with at least one other type of support, e.g., social support, decision support, or behaviour change support, can have positive effects on knowledge, social support, clinical, and behavioural outcomes.

However, high attrition rates impair the effectiveness of those online applications [12,13], and often health intervention websites are not often used more than once [14,15]. Since at the same time the impact of ehealth tools increases with usage rates [15,16], the intended outcome can be influence positively if users work longer and more intensely with the information provided [17,18] and use the website more often [19,20]. A preference-sensitive provision of information as well as an interactive presentation can lead to greater individualization and personalization which has been found to increase the usage and effectiveness of web-based health information tools [21,22]. The so-called concept of individual tailoring includes these strategies [23].

### Aims of the trial

The study evaluates an IHCA with information on chronic low back pain (CLBP), pain management education and decision support in a dialogue-based, tailored format against a standard website showing identical information without dialogue or tailoring. The primary hypothesis is that the dialogue-based, tailored format has larger effects on CLBP knowledge and patient empowerment than the standard website. Exploratory research questions are if usage is higher for the dialogue-based, tailored format and whether users feel better prepared for the consultation and, when facing a health decision, experience less decisional conflict after using the dialogue-based, tailored site rather than the standard website.

## Methods/Design

### Study design

**Figure 1:**
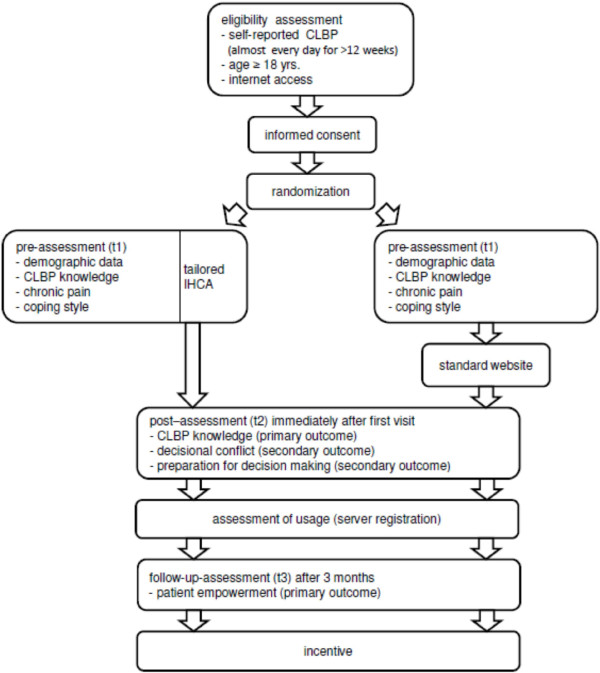
Study procedures.

In line with a comparable study [13] of our research group, a single-blinded two-armed randomised controlled trial (RCT) with a parallel design was chosen. Outcome measurements are planned before starting to use of the IHCA, after using the system and at three month follow-up. Knowledge about CLBP (primary outcome), decisional conflict, and preparation for decision making (secondary outcomes) are assessed immediately after using the system. Patient empowerment (secondary outcome) is assessed three months after the first visit (see Figure 1).

### Study procedures

First, the users are given an informed consent and have to fill out the pre-assessment (eligibility criteria, demographic data, chronic pain grade [14], coping style [15,16]). Subsequently, a random assignment of participants to the IHCA or the standard website is performed. During the dialogue of the system, the intervention group receives the questions necessary for tailoring. The participants who were allocated to the control condition without dialogue or tailoring complete these questionnaires just before entering the web page to control for disparities between control and intervention group at baseline. At the end of their first visit to the IHCA or the standard website all users are supposed to fill in the post-assessment.

Three months after starting to use the system, the participants of the study will receive an e-mail with a link to the follow-up online questionnaire. Non-monetary incentives can decrease attrition in online trials [17,18], we therefore decided that participants who have answered all questionnaires receive a coupon in the amount of 10 € for online shopping. Participants receive the code for this coupon at the end of the study by e-mail. Figure 1 provides an overview of the study procedures.

The intervention can be used as often and as long as participants intend, also between the post and follow-up assessment. Usage data on how often and how long participants have access to the web page is collected via server registrations. Usage data, questionnaire data, and personal data like e-mail addresses are stored separately. All data are pseudonymesed. The personal data will be erased after collecting the data. If a participant withdraws the informed consent for participation during the study procedure the data will be deleted forthwith. Otherwise, five years after the end of the study, the complete record of the study will be deleted. The study was approved by the Hamburg Medical Chamber ethics committee.

### Treatment allocation

Participants are informed within the informed consent that a random assignment to one of two website styles (dialogue-based, tailored vs standard) but with the same content will be made. The two web pages are not marked, consequently participants have no indications whether being in the control or intervention group. A specific software is used for randomisation.

### Recruitment

Health care for chronically ill people in Germany is divided into different sections, predominantly in in-patient rehabilitation centres and clinics for acute care, and out-patient primary care and specialist practices (orthopaedics, pain management). Health insurances mainly finance acute care in inpatient settings, primary care practices, and in specialist practices, whereas pension funds usually pay for treatment in rehabilitation clinics. As the inclusion of patients from all sectors was a primary study goal, patients were recruited over the whole spectrum of health care in Germany: via health insurance companies, pension funds, primary care and specialist practices, hospitals and rehabilitation centres, and self-help groups. Public dissemination strategies of the study included media such as newspapers, magazines, patient websites, and flyers. Further information on the study and the intervention can be found on the study website http://www.entscheidungshilfe.info.

### Study population

Eligibility criteria are age ≥ 18 years, access to the Internet, and self-reported CLBP (pain in the lower back almost every day for more than 12 weeks [19].

### Description of the intervention and control condition

The tailored IHCA as well as the standard website show essential information on CLBP (physiology of pain, acute vs. chronic pain, chronification, epidemiology, psychological aspects, coping and pain management) and related psychological problems (depression, anxiety), diagnostic procedures, and treatment options (pharmacological and non-pharmacological, see the list of the "IHCA's chapters and sections"). The website was designed similar in both conditions with regard to colours, typing, figures and pictures. All participants receives a password when signing in to the website via e-mail which can be used to log into the system as often as necessary.

### IHCA`s chapters & sections

•Introduction: What is this website?

• Where does the information on this site come from?

•CLBP Basics

• Physiological basics: back, spine, and intervertebral discs

• What exactly is pain?

• What is the difference between acute and chronic pain?

• Why does the pain stay when the physical injury heals?

• How many people live with CLBP?

• Managing CLBP in everyday life

•How is CLBP diagnosed?

• How much diagnostics makes sense and at which point?

• Diagnostic options

•How is CLBP treated?

• How much treatment makes sense and at which point?

• What is the natural, untreated course of CLBP?

• Are there accompanying conditions or sequelae of CLBP?

• Treatment options

•How do I recognize good treatment?

•Summary

•Additional information and literature

• Associations and self-help

• Web sites

• Journals

• Books

•Glossary

•Legal notice

•References

#### Intervention condition

The format of the website in the intervention condition tries to imitate a real conversation (dialogue-based) with a health professional, tailoring the content and tone of the dialogue to relevant patient characteristics. tunnelled design where the user proceeds through the content with a predetermined chronology was implemented in the intervention condition because such a design found to increase website use and knowledge gained from a website [20]. However, a tunnelled website might also evoke resistance [21], which is why we decided to give the user some degree of freedom over the chronology through the dialogue. Each information block ends with questions where the user can choose one of at least three reply options and receives a tailored answer. These tailored answers reflect the option the user has chosen using an empathic and appreciative intonation and build a personalized bridge to the next information block.

The concepts of coping style according to the avoidance-endurance model (AEM) [22] and health literacy (CLBP knowledge and preferred detailedness of information) were used for tailoring the provided information to individual preferences of the users. The individual coping style was assessed by using a questionnaire which is presented before starting the dialogue. There are four AEM subtypes: the “depressed endurer” (high endurance coping (EC) and high depressiveness (D)), the “happy endurer” (high EC and low D), the “depressed avoider” (low EC and high D), and the “adaptive coper” (low EC and low D; for examples see Table 1). During the virtual conversation, the content, the intonation and messages are tailored to the coping style of the individual user. This approach was inspired by Motivational Interviewing [23], a counseling technique used especially to address ambivalence about health behavior change.

**Table 1:** Tailoring to coping style

**Coping type**	**Adaptive coper**	**Happy endurer**	**Depressed endurer**	**Depressed avoider**
Description of coping style	You go about your pain in a matter-of-fact manner. You know that, on one hand, there is no serious disease behind it but that, on the other hand, they can signal you physical strain. You are good at making short breaks at the right time in order to keep up your daily routine – maybe temporarily a little slower than usual.	You tend to keep going in your daily routine even if pain is strong. This is, on one side, a personal strength. But at the same time you run the risk of actually straining your muscles, ligaments, joints and intervertebral discs.	You are a multitasker. Saying “No” to someone or not getting things done is hard on you. In order to meet requirements and get things done you push yourself to your limits and beyond. Often you don´t listen to your body before it is overstrained.	You are unsettled by your pain. You are worried that there might be a serious disease behind it, and / or you avoid activities that might increase the pain.
Take home message	Keep on like that! Make exercise part of your routine if you haven´t done it yet. Choose something fun and back-friendly. If you strengthen your muscles and stick to your relaxing breaks the pain should vanish soon.	Even if it´s hard: Try to pay more attention to your pain and get breaks early enough. Keep working, do things that are pleasant and fun, and keep moving – but remember to pause when you might need it!	Reconsider what you are asking from yourself: Do you really have to demand so much? Maybe there are times when it is possible to leave something undone, do it o.k. instead of perfect, or ask for assistance. These things are closely related to your pain.	Pain is unpleasant but not dangerous. Don´t let it suffocate you. Expand your limits step by step, and make pleasant activities a part of your everyday life.

**Figure 2:**
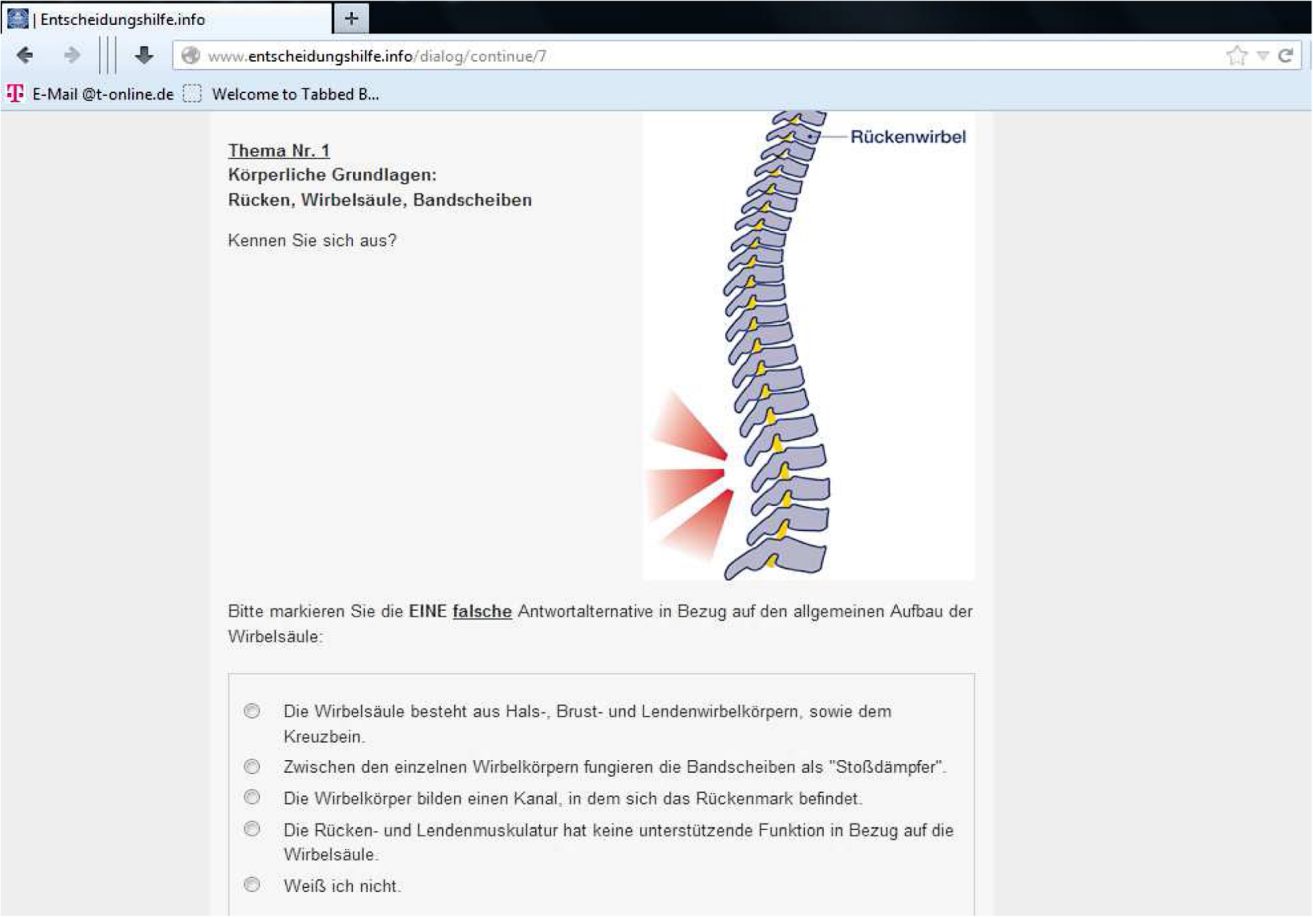
Dialogue window.

The items that assess CLBP knowledge are presented during the dialogue: In the beginning of the respective section (e.g. physiological basics), the user is questioned about his level of knowledge on this subject. Depending on the response, the subsequent section is accordingly amended. Figure 2 shows an exemplary dialogue window.

#### Control condition

**Figure 3:**
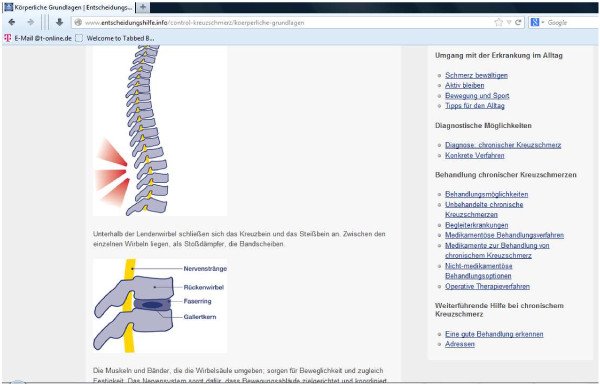
Control window.

For the control condition (using a standard website), no tailoring occurs for the provided information and the content is not offered using a predetermined chronology with a dialogue format and no guidance for the users throughout the content occurs. Using a classical webpage design, on each page a site map shows each content section which the user can chose by clicking on it to be forwarded to the selected content (see Figure 3).

### Potential risk for participants

Contraindications or side effects of IHCAs are not known.

### Intervention development and trial design

The intervention was developed in consideration of user-specific needs, the provided information is evidence-based. At the end of the development, a peer-review process was used to evaluate the system. First, a needs assessment was carried out to get insight into relevant topics for patients with CLBP. For this purpose, initially semi-structured interviews with five physicians (all specialized in orthopaedics) and nine patients with CLBP were conducted. Subsequently, we developed a questionnaire for potential users based on the results of the qualitative interviews, which should then be filled in by a new and larger patient sample (N = 117) with CLBP. The process and the results of needs assessment will be published in more detail elsewhere.

Primary sources for evidence-based information were treatment guidelines [19,24,25] and Cochrane Reviews [26-31]. In the course of developing the webpage, the selected health information was reviewed using an iterative process and interdisciplinary advisory group. After completion of the IHCA, four patients with CLBP and eight practitioners (5 orthopaedists, 1 neurologist, 1 medical journalist, and 1 psychologist) audited the system. Taking into account this feedback, the system was finalized and last modifications took place. The complete development process will be published in more detail elsewhere.

### Outcome assessment

CLBP knowledge (assessed after the first visit) and patient empowerment (assessed at three month follow-up) serve as primary outcomes. The level of knowledge about CLBP is tested with 29 statements about CLBP. The questions were developed in order to map the content covered in the sections of the IHCA. The questions can be answered with true / false / I don´t know. The Health Education Impact Questionnaire (HeiQ) [32,33] is used to assess patient empowerment. The HeiQ includes 42 items and 8 dimensions: Positive and Active Engagement in Life, Health Directed Behavior, Skill and Technique Acquisition, Constructive Attitudes and Approaches, Self-Monitoring and Insight, Health Service Navigation, Social Integration and Support, and Emotional Wellbeing. Schuler and colleagues [34] did the translation of the questionnaire into German and analysed the psychometric properties (Raykov’s Composite Reliability Coefficient, factorial and concurrent validity). The eight scales could be replicated, the questionnaire was found to be a reliable and valid measure. The dimension Social Integration and Support were removed since no effects of the IHCA were expected on these two dimensions and to keep the burden for the users at a minimum.

The shared decision-making concepts “decisional conflict” and “preparation for decision making” are used as secondary outcomes. Decisional conflict a state of uncertainty especially about medical decisions and is determined with the Decisional Conflict Scale (DCS) by O´Connor [35], a self-report instrument which measures personal verbalizations of uncertainty in choosing options. The questionnaire also measures factors contributing to uncertainty such as feeling uninformed, unclear about personal values and unsupported in decision making. It also assess the outcome of effective decision making with regard feeling the choice is informed, values-based, likely to be implemented and expressing satisfaction with the choice. The reliability of the measure is seen to be good with a Cronbach´s α between 0.78 and 0.92 [35], acceptable discriminant validity could be found. The Preparation for Decision Making Scale (PDMS) [36] is used for evaluating decision making processes relating to preparation for decision making. This 11 item scale assesses a patient´s view of how helpful a decision support intervention was in preparing for the next consultation with the practitioner and for making a treatment decision together with the practitioner. Reliability is very good ranging from α = .92 to α = .94. Both questionnaires are presented only to users who at the beginning of the survey stated that they probably will be making a treatment decision concerning their CLBP. In order to avoid missing data, all questionnaires include validation checks that alert participants when their answers are implausible or items are skipped.

### Statistical analyses

To empirically test the hypotheses, t-tests for independent samples will be calculated. We do not expect confounding factors due to randomizing the participants to the control and intervention condition and the most likely resulting structural equality of these two groups. In case baseline disparities should be detected, they will be taken account of as confounding variables in an analysis of covariance (ANCOVA). By using an intention-to-treat approach in which participants were analyzed in their original randomized groups regardless of the frequency or duration of website use we will include all randomized participants in the analyses in order to avoid biases such as non-random attrition of participants. Additionally we will perform a sensitivity analysis following the per-protocol approach. This will be done by including only participants that have completed all the measurements. A α level of ≤ 0.05 will utilized as the cutoff for statistical significance. With regard to the exploratory research questions, we expect only small sample sizes, hence only a small fraction of the participants will be confronted with the situation of having to make a treatment decision in the course their CLBP. Therefore, only these participants can be asked to fill in the DCS and PDMS. In order to be able to appraise the exactitude of testing, 95% confidence intervals will be defined for all parameters.

### Power calculation

On the basis of the Cochrane review by Murray et al. [37] we expect a small effect on the primary outcomes (Cohen’s d = 0.2). To detect a small effect with an α of 0.05 a power of 0.80 (one-tailed t-test), a sample size of N = 310 (155 per group) is required. Expecting a dropout 20% between registration and follow-up (3 months), we aim at including a sample of N = 414 at baseline.

## Discussion

In the current study, we are evaluating a tailored, dialogue-based online health information system that provides information on CLBP, accompanying psychological problems, diagnostic and treatment options, compared with a standard website which provides the same health information but without using a dialogue form, tailoring or interactive components. The IHCA for CLBP was developed extensively based on needs assessments, clinical practice guidelines and Cochrane Reviews. An interdisciplinary advisory committee and patients reviewed and revised the system. Knowledge on chronic low back pain and patient empowerment are used as primary outcomes of the randomized controlled trial. Secondary outcomes are decisional conflict, preparation for decision making, and website usage. This study is the first trial on a German language IHCA for people with chronic low back pain.

The study will have some limitations with regard to generalizability of the results. Probably most important, only people who have Internet access can take part in the study. However, 73% of the German population use the Internet regularly [38], but only 47% of the population over 50 years of age use the world wide web. Since CLBP becomes more prevalent with older age [39,40], this results in a high probability of leaving out a substantial part of our target population. This limits the generalizability of the results. It also impairs implementation and reach, and can be seen as a source for a systematic error in choosing the participants for this study.

Further limitations can arise from the use of online questionnaires, especially the relatively high nonresponse and attrition rates and concerns regarding data quality [41,42]. However, with regard to impairments of the quality of the data, there are indications that the psychometric properties of data collected via online questionnaire are at least as satisfactory as those obtained from printed measures [43,44]. Automatic validation checks of the survey software that alert participants when their answers are wrong or when items were not filled out can improved data quality [43]. Moreover, social desirability does not seem to be an equivalently important issue in online assessments compared to printed formats [45]. To account for non-responders we try to reduce attrition by reducing the length of the questionnaires and giving an incentive when all items and all questionnaires have been answered completely by the participant. The questionnaire assessing knowledge about chronic low back pain was newly developed for our purposes and is therefore not standardized measure, which bears another limitation concerning our measurements. However, the other used questionnaires (DCS, PDMS, HeiQ) are standardized. Finally, the questionnaire we use in our trial have not been evaluated or adapted for online use, which questions their transferability to findings when using printed versions [46].

## Abbreviations

CLBP: Chronic low back pain; SDM: Shared decision-making; IHCA: Interactive health communication application; RCT: Randomized controlled trial; PDMS: Preparation for decision making scale; DCS: Decisional conflict scale; HeiQ: Health education impact questionnaire; ANCOVA: Analysis of covariance.

## Competing interests

The authors declare that they have no competing interests.

## Authors’ contributions

JD participated in the conception and design of the study and drafted the manuscript. MH participated in the conception and design of the study and revised the manuscript. NW participated in the conception and design of the study and drafted the manuscript. All authors read and approved the final manuscript.

## Authors’ information

JD is a certified psychotherapist and a research associate at the Department of Medical Psychology. MH is a medical doctor, a certified psychotherapist and head of the Department of Medical Psychology. NW is a certified psychotherapist and a research associate at the Department of Medical Psychology.

## Pre-publication history

The pre-publication history for this paper can be accessed here:

http://www.biomedcentral.com/1472-6947/13/66/prepub
